# Investigating the association between cataract and the risk of herpes zoster in a cohort study

**DOI:** 10.3389/fmed.2025.1492365

**Published:** 2025-06-11

**Authors:** Shih-Wei Lai, Yu-Hung Kuo, Kuan-Fu Liao

**Affiliations:** ^1^School of Medicine, China Medical University, Taichung, Taiwan; ^2^Department of Family Medicine, China Medical University Hospital, Taichung, Taiwan; ^3^Department of Research, Taichung Tzu Chi Hospital, Taichung, Taiwan; ^4^College of Medicine, Tzu Chi University, Hualien, Taiwan; ^5^Division of Hepatogastroenterology, Department of Internal Medicine, Taichung Tzu Chi Hospital, Taichung, Taiwan

**Keywords:** cataract, cohort study, herpes zoster, Taiwan, varicella-zoster virus

## Abstract

**Objectives:**

This cohort study aimed to elucidate whether individuals with cataract are at an increased risk of herpes zoster in Taiwan.

**Methods:**

A cohort of individuals with cataract, aged 20–84 years, was assembled from electronic health records of Taiwan National Health Insurance Program spanning a period of 8 years (2013–2020). Those individuals who had visited an ophthalmology clinic but did not have a cataract diagnosis were selected as the non-cataract group from the same database. The incidence rate of herpes zoster within the follow-up period was calculated between the two groups. The risk of herpes zoster was compared between the two groups using a Cox proportional hazards model, adjusting for potential confounders.

**Results:**

The cohort study included 1,299,685 individuals in the cataract group and 1,138,887 individuals in the non-cataract group. The mean age was 64 years older and about 43.9% of study subjects were male in the cataract group. The mean age was 63.6 years older and about 47.1% of study subjects were male in the non-cataract group. The incidence rate of herpes zoster was 10.84 per 1,000 person-years in the cataract group and 8.64 per 1,000 person-years in the non-cataract group (incidence rate ratio = 1.25, 95% CI = 1.24–1.27, *P* < 0.001). After adjusting for potential confounders, the hazard ratio was 1.22 for herpes zoster in individuals with cataract when compared with those without cataract (95% CI = 1.21–1.23, *P* < 0.001).

**Conclusion:**

Our cohort study reveals that individuals with cataract are at an increased risk of developing herpes zoster. Our findings highlight the importance of considering the increased susceptibility to herpes zoster in individuals with cataract and the potential benefits of preventive measures such as herpes zoster vaccination.

## Introduction

Cataract and herpes zoster are indeed prevalent conditions among the aging population and represent significant public health challenges worldwide due to their high prevalence, associated morbidity, and healthcare burden. Cataract is characterized by the progressive clouding of the lens that is associated with decreased visual acuity. Cataract is still the leading cause of blindness and vision impairment. (1–3). In 2020, approximately 100.5 million people worldwide had blindness or moderate to severe vision impairment due to cataract, and this number has increased over the past 30 years (4). The prevalence of cataract increases markedly with age, with a higher prevalence observed in older age groups. For example, in a meta-analysis conducted by Hashemi et al., the overall prevalence of cataract was 3.01% in ages 20–39, 16.97% in ages 40–59, and 54.38% in ages 60 or older, respectively (5). This is consistent with the well-established understanding that aging is a risk factor for the development of cataract.

Herpes zoster results from the reactivation of the varicella-zoster virus (VZV), which remains dormant in the sensory nerve ganglia after primary infection with varicella (chickenpox) (6–8). Herpes zoster causes painful manifestations and subsequent impairment in quality of life, so it is a major health burden. The reactivation of VZV typically occurs later in life or occurs in other conditions that compromise the immune system (9–11). While cell-mediated immunity to varicella-zoster virus declines with age, the risk of developing herpes zoster increases with age, especially after age 50 (12–14). This decline in immunity is thought to be one of the key factors contributing to the increased risk of developing herpes zoster, especially in older adults. For example, the incidence of herpes zoster for people aged 50–59 years was 3.59 per 1,000 person-years and peaked at 9.94 per 1,000 person-years for people aged ≥80 years in a population-based study conducted by Södergren et al in Sweden (15).

While both conditions share some risk factors, such as aging and impaired immune status (5, 15–17), the relationship between cataract and herpes zoster remains unexplored. Understanding the potential link between cataract and herpes zoster is of importance from both public health and clinical perspectives. Elucidating this association could provide preventive strategies aimed at reducing the burden of herpes zoster among individuals with cataract, particularly through specific vaccination. Therefore, the present cohort study aimed to fill this knowledge gap by systematically investigating whether individuals with cataract are at increased risk of developing herpes zoster compared with those without cataract. Through comprehensive data analysis, we seek to provide valuable insights into the relationship between these two common age-related diseases to inform public health strategies and clinical practice in Taiwan.

## Materials and methods

### Data source

We utilized claims data spanning from 2013 to 2020 from the Taiwan National Health Insurance Program as our data source. Claims data contain comprehensive records of medical services utilized by beneficiaries, including outpatient visits, inpatient admissions, emergency room visits, and medication prescriptions.

### Study subjects and study design

**FIGURE 1:**
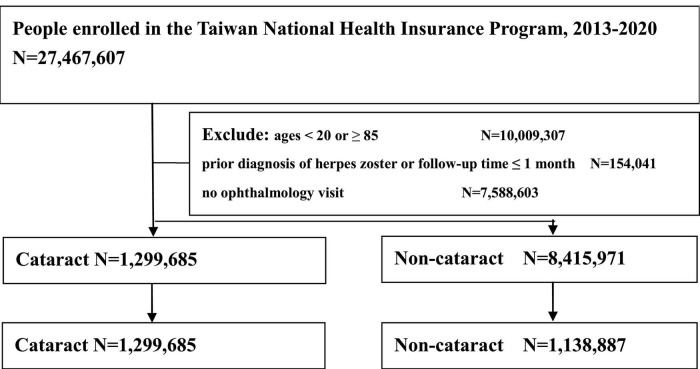
Algorithmic approach to selection of study subjects.

**FIGURE 2:**
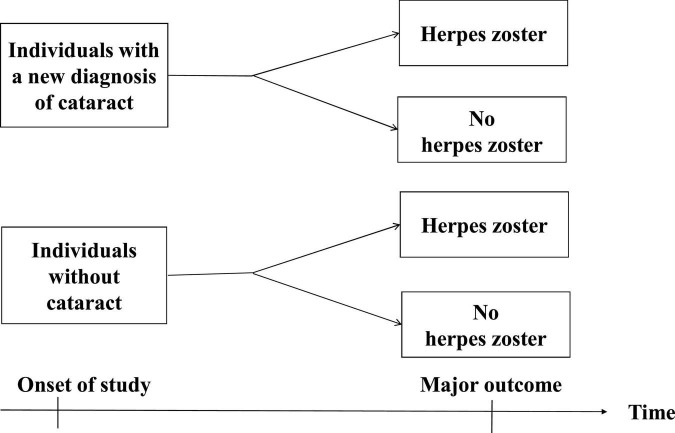
Time frame of the cohort study.

The cataract group included individuals aged 20 years or older with a new diagnosis of cataract (based on the International Classification of Diseases 9*^th^* and 10*^th^* Revisions, ICD-9 codes and ICD-10 codes). The index date was defined as the date of diagnosing cataract. Those individuals who had visited an ophthalmology clinic but did not have a cataract diagnosis were randomly selected as the non-cataract group. The index date for the non-cataract group was defined as the date of their first outpatient visit in 2013. During matching process, we found there were more individuals with cataract than those without cataract among individuals aged over 60, making it difficult to match the cataract and non-cataract groups for comparison. Therefore, for individuals under 60 years old, the cataract and non-cataract groups were matched by sex and age. However, the cataract and non-cataract groups were not matched by sex and age for individuals aged 60 and above. To establish the temporal relationship, individuals with a diagnosis of herpes zoster prior to the index date were excluded from the study. Additionally, individuals with a follow-up period of less than one month were also excluded to ensure sufficient observation time (Figure 1). Then a population-based, retrospective cohort study was designed to follow individuals over time to assess the primary outcome (Figure 2).

### Comorbidities

Some comorbidities before the index date were selected as covariables in statistical analyses to adjust for potential confounding effects, including alcohol-related disease, cerebrovascular disease, chronic obstructive pulmonary disease, coronary artery disease, diabetes mellitus, hyperlipidemia, and hypertension, all of which were relevant to many health outcomes. These comorbidities were diagnosed based on ICD-9 codes and ICD-10 codes.

### Primary outcome

The primary outcome of the cohort study was defined as the occurrence of a new diagnosis of herpes zoster among the study subjects during the follow-up period (based on ICD-9 codes 053 and ICD-10 codes B02). All study subjects were monitored until the occurrence of a new diagnosis of herpes zoster or until the end of the cohort period in 2020.

### Statistical analysis

If the *P*-value was less than 0.05, the null hypothesis was rejected, indicating that the observed results were statistically significant. Chi-square test was used to compare baseline characteristics of categorical variables between individuals with cataract and individuals without cataract. Student’s *t*-test was used to compare baseline characteristics of continuous variables between individuals with cataract and individuals without cataract. Incidence rate of herpes zoster was calculated as the number of new cases of herpes zoster identified during the follow-up period divided by the total person-years during the same period. The Kaplan-Meier curve was constructed to illustrate the cumulative incidence of herpes zoster among individuals with cataract and individuals without cataract. Subsequent statistical analysis by the log-rank test was conducted to assess whether there were significant differences in the cumulative incidence of herpes zoster between the two groups over the follow-up period. The Cox proportional hazards regression model was used to estimate hazard ratio (HR) and corresponding 95% confidence interval (CI) for the risk of developing herpes zoster in individuals with cataract compared with individuals without cataract, adjusting for potential confounding variables. The assumption of proportional hazards in a Cox proportional hazards regression model was assessed using a test of scaled Schoenfeld residuals. The assumption was not violated in the study. In other words, the hazard ratio estimated by the Cox model could be considered valid. The SAS software was employed in all analyses of the study (version 9.4 for Windows; SAS Institute Inc., Cary, NC, United States).

## Results

### Basic information of study subjects

In Table 1, the cohort study included 1,299,685 individuals in the cataract group and 1,138,887 individuals in the non-cataract group. The mean age was 64 years older and about 43.9% of study subjects were male in the cataract group. The mean age was 63.6 years older and about 47.1% of study subjects were male in the non-cataract group. The proportions of alcohol-related disease, chronic obstructive pulmonary disease, coronary artery disease, diabetes mellitus, hyperlipidemia, and hypertension were higher in the cataract group than the non-cataract group, resulting in statistically significant differences (Chi-square test, *P* < 0.001), but the proportion of cerebrovascular disease was higher in the non-cataract group than the cataract group, resulting in statistically significant differences (Chi-square test, *P* < 0.001).

**TABLE 1:** Baseline information between cataract group and non-cataract group.

Variable	Cataract		Non-cataract		*P* value*
	*N* = 1,299,685		*N* = 1,138,887		
Sex, *n* (%)					<0.001
Male	570880	43.9	536284	47.1	–
Female	728805	56.1	602603	52.9	–
Age group (years), *n* (%)					<0.001
20–39	14,978	1.2	14,978	1.3	–
40–64	668,245	51.4	684,035	60.1	–
65–84	616,462	47.4	439,874	38.6	–
Age (years), mean ± standard deviation^†^	64.0 ± 9.3		63.6 ± 10.3		<0.001
Follow-up time (years) (median, IQR)^†^	4.8	2.8–6.5	7.9	7.9–7.9	<0.001
**Baseline comorbidities**					
Alcohol-related disease, *n* (%)	7,527	0.6	4,787	0.4	<0.001
Cerebrovascular disease, *n* (%)	87,025	6.7	83,469	7.3	<0.001
Chronic obstructive pulmonary disease, *n* (%)	96,167	7.4	81,591	7.2	<0.001
Coronary artery disease, *n* (%)	151,439	11.7	120,346	10.6	<0.001
Diabetes mellitus, *n* (%)	340,153	26.2	198,025	17.4	<0.001
Hyperlipidemia, *n* (%)	393,000	30.2	223,500	19.6	<0.001
Hypertension, *n* (%)	572,113	44.0	434,859	38.2	<0.001

Data are presented as the number of subjects in each group, with percentages given in parentheses.

*Chi-square test.

^†^*t*-test comparing subjects with and without cataract. IQR: interquartile range.

### Incidence density of herpes zoster

In Table 2, the incidence rate of herpes zoster was 10.84 per 1,000 person-years in the cataract group. In contrast, the incidence rate of herpes zoster was 8.64 per 1,000 person-years in the non-cataract group. The cataract group had a higher incidence rate of herpes zoster compared with the non-cataract group (incidence rate ratio = 1.25, 95% CI = 1.24–1.27, *P* < 0.001). After stratification by sex and age groups, the cataract group exhibited a higher incidence rate of herpes zoster compared with the non-cataract group. Specifically, the incidence ratio of herpes zoster was 1.27 in males with cataract compared with males without cataract, and 1.23 in females with cataract compared with females without cataract. Additionally, among age groups, the incidence ratio of herpes zoster was 1.56 in individuals aged 20–39 with cataract compared with those without cataract, 1.17 in individuals aged 40–64 with cataract compared with their non-cataract counterparts, and 1.29 in individuals aged 65–84 with cataract compared with those without cataract.

**TABLE 2:** Incidence density of herpes zoster between subjects with and without cataract stratified by sex and age.

	Cataract				Non-cataract						
Variable	*N*	Event	Person-years	Incidence^†^	*N*	Event	Person-years	Incidence^†^	IRR^#^	(95% CI)	*P*-value
All	1299,685	64,625	595,9214	10.84	1,138,887	75,184	8,699,890	8.64	1.25	(1.24–1.27)	<0.001
**Sex**											
Male	570,880	25,712	2,582,972	9.95	536,284	32,200	4,109,170	7.84	1.27	(1.25–1.29)	<0.001
Female	728,805	38,913	3,376,242	11.53	602,603	42,984	4,590,720	9.36	1.23	(1.21–1.25)	<0.001
**Age group (years)**											
20–39	14,978	244	65,466	3.73	14,978	281	117,277	2.40	1.56	(1.31–1.85)	<0.001
40–64	668,245	29,348	3049,079	9.63	684,035	42,976	524,1608	8.20	1.17	(1.16–1.19)	<0.001
65–84	616,462	35,033	2,844,669	12.32	439,874	31,927	3,341,005	9.56	1.29	(1.27–1.31)	<0.001

^†^Incidence: per 1,000 person-years. ^#^IRR (incidence rate ratio): cataract vs. non-cataract (95% confidence interval).

**FIGURE 3:**
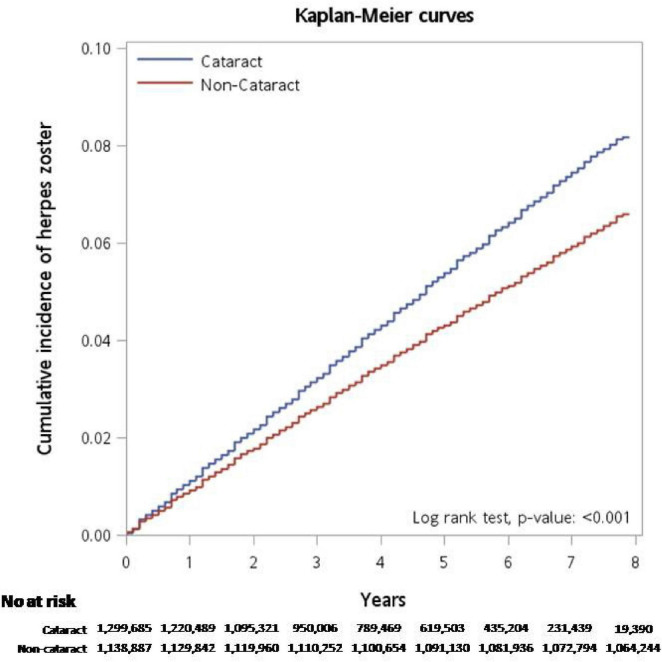
Kaplan-Meier curve reveals that the cumulative incidence of herpes zoster was higher in the cataract group compared with the non-cataract group during the cohort period (*P* < 0.001).

In Figure 3, the Kaplan-Meier curve reveals that the cumulative incidence of herpes zoster was higher for the cataract group compared with the non-cataract group during the cohort period (*P* < 0.001). It indicates a statistically significant difference between the cataract and non-cataract groups.

### Association of herpes zoster with cataract and co-variables

In Table 3, after adjusting for potential confounders including sex, age, alcohol-related disease, cerebrovascular disease, chronic obstructive pulmonary disease, coronary artery disease, hyperlipidemia, and hypertension, the Cox proportional hazards regression model showed the hazard ratio (HR) for herpes zoster in the cataract group compared with the non-cataract group was 1.22 (95% CI = 1.21–1.23, *P* < 0.001). The HR serves as reliable measures of the strength of these associations and it suggests a significant positive association between cataract and the risk of developing herpes zoster, indicating an increased likelihood of herpes zoster among individuals with cataract. The 95% CI of 1.21–1.23 reflects the precision of the estimate, providing a range within which we can be 95% confident that the true hazard ratio lies, suggesting a strong association between cataract and herpes zoster risk.

**TABLE 3:** Multivariable cox model measuring hazard ratio and 95% confidence interval for herpes zoster associated with cataract and co-variables.

	Crude			Adjusted^†^		
Variable	HR	(95% CI)	*P*-value	HR	(95% CI)	*P*-value
Sex (male vs. female)	0.84	0.83–0.85	<0.001	0.85	0.84–0.86	<0.001
**Age group (years)**						
20–39	Ref.	–	–	Ref.	–	–
40–64	3.02	2.77–3.29	<0.001	2.87	2.64–3.13	<0.001
65–84	3.75	3.44–4.08	<0.001	3.41	3.13–3.72	<0.001
Cataract (yes vs. no)	1.27	1.25–1.28	<0.001	1.22	1.21–1.23	<0.001
**Baseline comorbidities (yes vs. no)**						
Alcohol-related disease	0.74	0.68–0.81	<0.001	0.79	0.73–0.87	<0.001
Cerebrovascular disease	0.96	0.94–0.98	<0.001	0.88	0.86–0.90	<0.001
Chronic obstructive pulmonary disease	1.24	1.21–1.26	<0.001	1.18	1.16–1.20	<0.001
Coronary artery disease	1.15	1.14-1.17	<0.001	1.05	1.03–1.07	<0.001
Diabetes mellitus	1.00	0.99–1.01	1.000	–	–	–
Hyperlipidemia	1.19	1.17–1.20	<0.001	1.10	1.09–1.12	<0.001
Hypertension	1.16	1.15–1.18	<0.001	1.05	1.04–1.07	<0.001

^†^Adjusted for sex, age, alcohol-related disease, cerebrovascular disease, chronic obstructive pulmonary disease, coronary artery disease, hyperlipidemia, and hypertension.

## Discussion

In this present cohort study, we found that the overall incidence rates of herpes zoster were higher in individuals with cataract compared with their respective controls across all three age groups (20–39, 40–64, and 65–84). Even though herpes zoster is typically more common among older adults, there is still a significantly higher incidence ratio of herpes zoster observed in individuals aged 20–39 who have cataract compared with those without cataract (Table 2). This suggests that cataract may be associated with an increased risk of herpes zoster across different age groups, including younger adults. After adjusting for potential confounders such as age, sex, and comorbidities, we found that individuals with cataract had a 22% increased hazard of experiencing herpes zoster over time compared with those without cataract. This present study represents a novel contribution to the literature as there are no previous studies examining the association between cataract and the risk of herpes zoster. Our study fills a gap in the literature by conducting the first investigation of this issue.

At present, cataract itself may not be the sole factor driving the increased risk of herpes zoster. Instead, this association could result from a combination of factors, including age-related immune system changes, comorbidities, shared risk factors, and underlying health conditions. In our study, individuals aged 20–39 accounted for only 1.2% of all individuals with cataract, indicating that cataract predominantly affects older adults. This finding aligns with a meta-analysis by Hashemi et al., which reported an overall prevalence of cataract of 3.01% in individuals aged 20–39 (5). Therefore, older adults with cataract might exhibit age-related immune dysfunction, particularly a decline in varicella-zoster virus-specific T cell-mediated immunity, making them more susceptible to the development of herpes zoster (18–21). The cataract group had a higher proportion of diabetes mellitus compared with the non-cataract group (26.2% versus 17.4%). Diabetes mellitus could potentially link the association between cataract and herpes zoster (22–25). However, it is important to note that while diabetes mellitus can increase the risk, it is not necessarily a direct cause of either cataract or herpes zoster.

Several limitations should be addressed in this cohort study to ensure the validity and reliability of the findings. First, there might be inherent biases in the selection of study subjects, potentially affecting the generalizability of the results, Second, the observed association between cataract and an increased probability of herpes zoster could be influenced by unmeasured confounding factors that were not fully accounted for in our study. For example, alcohol consumption, smoking history, socioeconomic status, behavioral factors, increased healthcare utilization, and other underlying health conditions might influence the development of cataract and herpes zoster. However, we included alcohol-related disease as a proxy for alcohol consumption and chronic obstructive pulmonary disease as a proxy for smoking history. These proxies help minimize the influence of these confounders on the results, thereby strengthening the validity of our study findings. Third, variability in the measurement of cataract and herpes zoster could introduce measurement error, potentially biasing the estimates of association. However, based on the good quality of medical care in Taiwan, the likelihood of measurement error in the diagnosis of conditions like cataract and herpes zoster can be minimized. Fourth, when interpreting the study results, it is essential to consider that the characteristics of the study population, such as individual health conditions and demographics, and geographic location, may limit the generalizability of our findings to other populations. The incidence of cataract and herpes zoster can vary due to regional, ethnic, genetic, and environmental differences, as well as variations in healthcare access and lifestyle factors. Future studies conducted in diverse populations are needed to validate our findings and to explore potential regional or ethnic variations in the association between cataract and herpes zoster. Fifth, there might be a potential misclassification; therefore, we defined the non-cataract group as individuals who had visited an ophthalmology clinic but were not diagnosed with cataract. This approach minimizes the likelihood of undiagnosed cataract cases within the non-cataract group. Sixth, there were more individuals with cataract than those without cataract among individuals aged over 60 in this study, making it difficult to match the cataract and non-cataract groups for comparison. It could introduce bias. However, we adjusted for potential confounders in the statistical models to account for baseline differences between the groups. Our analytical approach helps minimize potential biases and strengthens the validity of our results. Seventh, individuals in the cataract group may differ from those in the non-cataract group in terms of their tendency to seek medical care and engage in other healthy behaviors. Once diagnosed with cataract, these individuals are likely to visit the doctor regularly (e.g., every 6 months to a year), which increases the likelihood of detecting non-vision-threatening conditions such as herpes zoster. This increased medical surveillance could contribute to the observed association between cataract and herpes zoster. Despite the limitations mentioned earlier, there were several strengths associated with the study. First, a large sample size improves the statistical power of the study, enhances the precision of estimates, and increases the ability to detect differences between the cataract and non-cataract groups. Second, a long follow-up duration of 8 years allowed for the observation of outcomes over an extended period, providing a comprehensive understanding of the relationship between cataract and herpes zoster. Third, the novel findings provide updated insights into previously unexplored research areas. Fourth, employing rigorous study design and statistical analysis methods can minimize bias and improve the validity of the study.

## Conclusion

Our population-based, retrospective cohort study reveals that individuals with cataract have a higher incidence of herpes zoster compared with those without cataract across different age groups, including younger adults. After adjusting for potential confounding variables, individuals with cataract remain at an increased risk of developing herpes zoster. Our findings highlight the importance of considering the increased susceptibility to herpes zoster among individuals with cataract. Public health policies should consider offering herpes zoster vaccination to this population. Given the absence of directly comparable studies, we suggest that further research is needed to explore whether the relationship between cataract and herpes zoster is merely a coincidental association or a direct correlation.

## Data Availability

The raw data supporting the conclusions of this article will be made available by the authors, without undue reservation.
